# Targeting Parthanatos in Ischemic Stroke

**DOI:** 10.3389/fneur.2021.662034

**Published:** 2021-05-05

**Authors:** Raymond C. Koehler, Valina L. Dawson, Ted M. Dawson

**Affiliations:** ^1^Department of Anesthesiology and Critical Care Medicine, The Johns Hopkins University, Baltimore, MD, United States; ^2^Neuroregeneration and Stem Cell Programs, The Institute of Cell Engineering, The Johns Hopkins University, Baltimore, MD, United States; ^3^Department of Neurology, The Johns Hopkins University, Baltimore, MD, United States; ^4^Department of Neuroscience, The Johns Hopkins University, Baltimore, MD, United States; ^5^Department of Physiology, The Johns Hopkins University, Baltimore, MD, United States; ^6^Department of Pharmacology and Molecular Sciences, The Johns Hopkins University, Baltimore, MD, United States

**Keywords:** cerebral ischemia, neuroinflammation, parthanatos, poly(ADP ribose) polymerase, stroke, middle cerebral artery occlusion

## Abstract

Parthanatos is a cell death signaling pathway in which excessive oxidative damage to DNA leads to over-activation of poly(ADP-ribose) polymerase (PARP). PARP then generates the formation of large poly(ADP-ribose) polymers that induce the release of apoptosis-inducing factor from the outer mitochondrial membrane. In the cytosol, apoptosis-inducing factor forms a complex with macrophage migration inhibitory factor that translocates into the nucleus where it degrades DNA and produces cell death. In a review of the literature, we identified 24 publications from 13 laboratories that support a role for parthanatos in young male mice and rats subjected to transient and permanent middle cerebral artery occlusion (MCAO). Investigators base their conclusions on the use of nine different PARP inhibitors (19 studies) or PARP1-null mice (7 studies). Several studies indicate a therapeutic window of 4–6 h after MCAO. In young female rats, two studies using two different PARP inhibitors from two labs support a role for parthanatos, whereas two studies from one lab do not support a role in young female PARP1-null mice. In addition to parthanatos, a body of literature indicates that PARP inhibitors can reduce neuroinflammation by interfering with NF-κB transcription, suppressing matrix metaloproteinase-9 release, and limiting blood-brain barrier damage and hemorrhagic transformation. Overall, most of the literature strongly supports the scientific premise that a PARP inhibitor is neuroprotective, even when most did not report behavior outcomes or address the issue of randomization and treatment concealment. Several third-generation PARP inhibitors entered clinical oncology trials without major adverse effects and could be repurposed for stroke. Evaluation in aged animals or animals with comorbidities will be important before moving into clinical stroke trials.

## Introduction

With the recent success of endovascular thrombectomy in establishing recanalization and, in many cases, reperfusion in stroke patients with large vessel occlusions, neurologic outcome has been improved, especially for those who have sufficient collateral blood flow to slow the transition from penumbra to irreversible injury (1–7). However, a significant portion of those with successful reperfusion may still die or be left with significant neurologic deficits. Thus, interest has been renewed in finding neuroprotective agents that can be used as an adjunct with thrombectomy and clot lysis (8–10).

One potential target that has been investigated in the context of cerebral ischemia over the past three decades is poly(ADP-ribose) polymerase (PARP). This family of enzymes is involved in post-translational protein modification by ADP-ribosylation. PARP1, in particular, is well-known for its role in the DNA repair process. Indeed, prolonged inhibition of PARP1 is cytotoxic and has been used as a strategy in oncology to induce cell death in rapidly proliferating cancer cells. However, when numerous DNA strands are damaged and PARP activity reaches high levels, this elevated activity can induce programmed cell death. This PARP-dependent form of programmed cell death has been termed parthanatos. A number of PARP inhibitors have been developed that block parthanatos, including that which occurs in neurons in response to excitotoxic stimuli and oxygen-glucose deprivation (OGD). A body of literature indicates that, in addition to arresting cell death, PARP inhibitors can reduce the NFκB-mediated pro-inflammatory response of microglia, suppress matrix metalloproteinase (MMP-9) release, and limit blood-brain barrier (BBB) damage and hemorrhagic transformation. Thus, PARP inhibitors can act to directly protect neurons and to limit secondary injury from inflammatory processes. These multi-targeted actions of PARP inhibitors make them strong candidates for prolonging neuronal viability and ameliorating reperfusion injury. In this review, we will consider the mechanisms by which PARP activity contributes to cell death and neuroinflammation and then examine the evidence that PARP inhibitors can protect the brain from ischemic stroke.

## Mechanisms of PARP-Dependent Neuronal Cell Death (Parthanatos)

Damage to DNA by oxidants such as peroxynitrite rapidly activates PARP (11, 12). Peroxynitrite formation is increased by the excess production of nitric oxide (NO) and superoxide anion (13–15). In the context of cerebral ischemia, reduced ATP production results in neuronal depolarization, release of synaptic glutamate, and decreased reuptake by glutamate transporters (16, 17). Excess extracellular glutamate activates N-methyl-D-aspartate (NMDA) and other glutamate receptors, leading to an excitotoxic process that involves increased intracellular Ca^2+^. A key aspect of the excitotoxic process is the association of the NR2B subunit to post-synaptic density-95 scaffolding protein and stimulation of NO synthase (18). NO is highly diffusible and can target other neurons and cell types. Major sources of superoxide during and after ischemia are the uncoupling of the mitochondrial electron transport chain (19) and the activation of NADPH oxidases (20, 21). In the context of reperfusion, the increased reoxygenation supports excess superoxide production (22). Although regeneration of ATP can reduce extrasynaptic glutamate detected by microdialysis, it is thought that increased glutamate levels in the extracellular synaptic cleft can persist during reperfusion (23, 24). In addition, altered phosphorylation of NMDA receptor subunits and of neuronal nitric oxide synthase (nNOS) can sustain the activity of these molecules through their persistent association with post-synaptic density proteins during the reperfusion period (25). Thus, peroxynitrite formation can be augmented during the reperfusion period and lead to further DNA damage and activation of PARP. This scenario provides a basis for the therapeutic potential of PARP inhibitors administered during reperfusion produced by thrombectomy and tissue plasminogen activator administration.

**Figure 1:**
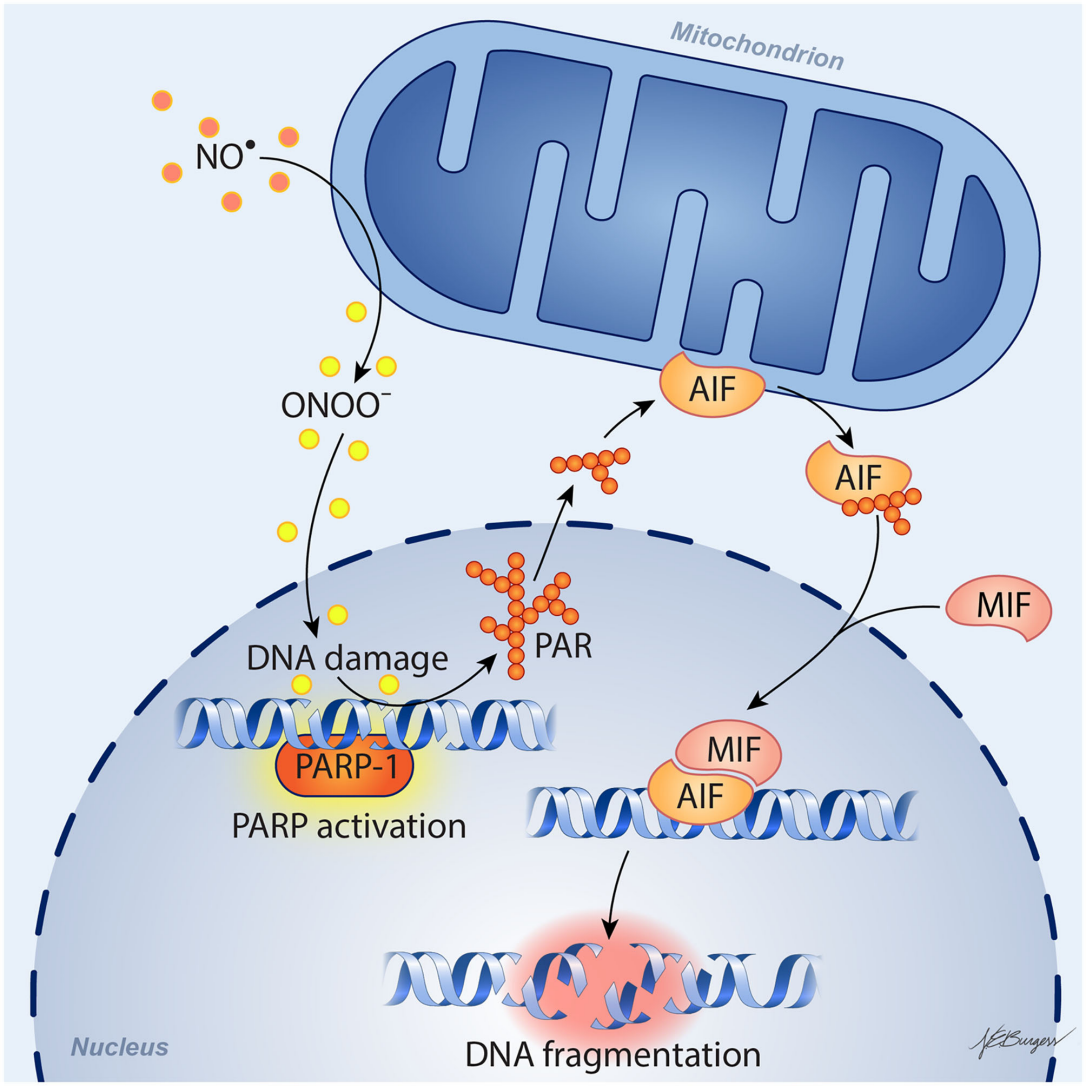
Schematic diagram of parthanatos signaling. Activation of NMDA receptors stimulates neuronal nitric oxide (NO) synthase (nNOS). Ischemia and reperfusion lead to excess superoxide production in mitochondria and from NADPH oxidase. The abundant NO and superoxide spontaneously generate peroxynitrite (ONOO^−^). This strong prooxidant, along with other reactive oxygen species (ROS), damages DNA strands and thereby causes activation of poly(ADP-ribose) polymerase (PARP). When the DNA damage is great, the consequent over-activation of PARP leads to abundant PAR polymer formation in the nucleus; some of the poly(ADP) ribosylated carrier proteins exit the nucleus and cause the release of apoptosis-inducing factor (AIF) from a pool on the outer mitochondrial membrane. Once in the cytosol, AIF can bind to macrophage migration inhibitory factor (MIF). Together, they enter the nucleus and produce large-scale DNA degradation and cell death.

In 1994, the Dawson laboratory reported that exposing cultured mouse neurons to NMDA or high levels of NO activates PARP and that inhibition of PARP protects the neurons from cell death (26). Moreover, PARP1^−/−^ and nNOS^−/−^ murine neurons were shown to be resistant to cell death evoked by NMDA (27, 28). These findings have more recently been replicated in human neuronal cultures derived from embryonic stem cells and inducible pluripotent stem cells, as these cultures were protected from NMDA and OGD by inhibition of PARP or nNOS (29). Importantly, protective effects were seen in both male and female human neurons with the new generation of PARP inhibitors developed for use in oncology: olaparib, veliparib, rucaparib, and talazoparib. PARP's instigation of cell death involves release of apoptosis-inducing factor (AIF) from a mobile pool in the outer mitochondrial membrane (30–32). This release requires the formation of large PAR polymers by PARP and the binding of PAR to a PAR-binding site motif on AIF (33). Degradation of the PAR polymer by PAR glycohydrolase (PARG) decreased the release of AIF by PAR polymers (34) and by NMDA (35). Once AIF is released from the mitochondria, it can bind to macrophage migration inhibitory factor (MIF) (36). The binding of AIF to MIF results in uptake of the complex into the nucleus where the complex binds to DNA. The binding enables MIF to act as an endonuclease that degrades genomic DNA into 20–50 kb DNA fragments, and that leads to cell death. This signaling pathway is summarized in Figure 1 and is reviewed in detail elsewhere (37).

Prolonged ischemia results in classical necrosis, whereas reperfusion can result in sustained restitution of cell function if the duration and severity of ischemia are not prolonged or intense. With reperfusion after an intermediate ischemic duration and/or intermediate ischemic severity, some neurons can generate sufficient ATP to restore membrane potential. Often, these neurons then undergo various forms of delayed programmed cell death. Delayed apoptosis may occur in ischemic border regions where ischemia is less severe and not as prolonged. However, with durations of middle cerebral artery (MCA) occlusion (MCAO) lasting >30 min, programmed necrosis predominates. The two most studied forms of programmed necrosis in stroke are parthanatos and RIP1-dependent necroptosis. The latter involves formation of a necroptosome consisting of phosphorylated RIP-1 and phosphorylated RIP-3 anchored by MLKL. Inhibition of necroptosome formation by intraventricular injection of necrostatin-1 decreases infarct volume by one-third (38), but clinical translation of this work is limited by the poor BBB penetration of this inhibitor class. In contrast, several different PARP inhibitors have proven effective following systemic administration, yielding a reduction in infarct volume that often exceeds 50%, as reviewed below.

In addition to the parthanatos mechanism of neurodegeneration described above, the product of PARP activation, PAR, is emerging as an important contributor in the separation and aggregation of amyloid proteins in the degenerative process of Parkinson's disease, Alzheimer's disease and amyotrophic lateral sclerosis (39). Future studies investigating the role of PAR dependent liquid-liquid phase separation and aggregation of proteins are needed to determine whether this plays a role in stroke pathogenesis.

## Mechanisms of PARP-Dependent Neuroinflammation

In addition to acting directly on neurons to block parthanatos, PARP inhibitors can exert anti-inflammatory effects and protect endothelial cells. For example, PARP inhibitors have been reported to exert anti-inflammatory effects in a wide range of systemic models of inflammation (endotoxemia, peritonitis, colitis, streptozotocin-induced diabetes) (40) and to protect endothelial-dependent responses from oxidant stress associated with aging (41), streptozotocin-induced diabetes (42), hypochlorite (43), and H_2_O_2_ (44). Exposing macrophages to oxidants stimulates their PARP activity (45). Exposing cultured BV2 microglial cells to lipopolysaccharide induces NF-κB, inducible nitric oxide synthase (iNOS), nitrite formation, reactive oxygen species formation, and tumor necrosis factor-α (TNFα), all of which are attenuated by the PARP inhibitor PJ34 (46). With global cerebral ischemia, PJ34 pre-treatment attenuated loss of tight junctions, Evan's Blue extravasation, cerebral edema, and neutrophil infiltration (47). Microglia-mediated loss of tight junctions is absent in co-cultures of PARP1-null microglia and endothelial cells (48). In cultured human brain microvascular endothelial cells, PARP inhibitors reduce AIF nuclear translocation and cell death after repeated bouts of hypoxia and reoxygenation (49). In microglial cultures exposed to the alarmin S100B, induction of interleukin-1β (IL-1β) and iNOS was blunted by the PARP inhibitor veliparib, and injection of S100B directly into striatum produced an accumulation of Iba1-positive microglia that was attenuated by veliparib (50). Glutamate activation of microglia is also PARP-dependent (51).

**Figure 2:**
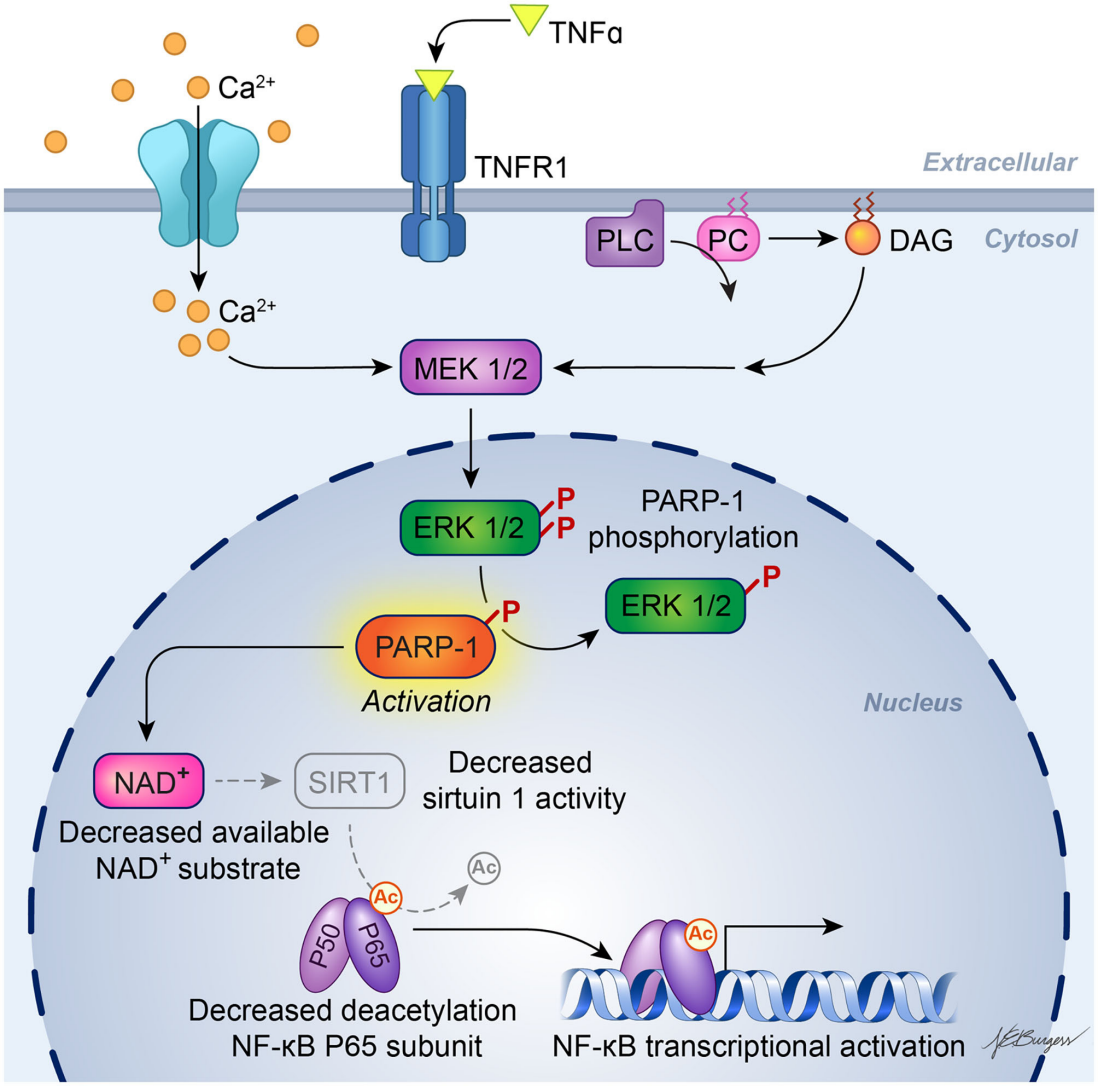
Schematic diagram of PARP activation in inflammatory signaling. Experiments in cultured microglia and astrocytes indicate that binding of TNFα to its receptor, TNFR1, leads to calcium influx and activation of phosphatidylcholine (PC)-specific phospholipase C (PC-PLC), resulting in formation of diacylglycerol (DAG) and downstream action of the MEK1/2-ERK1/2 pathway. ERK2 phosphorylation of PARP-1 increases PARP-1 activity, which then facilitates activation of NF-κB transcription. One possible mechanism whereby PARP-1 facilitates NF-κB transcription is by depleting NAD^+^, which is also used as a substrate by sirtuin-1. Sirtuin-1 normally keeps the P65 subunit of NF-κB deacetylated, but a decrease in sirtuin-1 activity will result in acetylated P65, which enables NF-κB transcription. [Adapted, with permission, from Vuong et al. (54) and Kaupinnen et al. (56)].

It is known that enzymatic activity of PARP1 promotes NF-κB–driven transcription in microglia (52). The underlying mechanism has been investigated by Kauppinen et al. In microglia, PARP1 activity facilitates NF-κB binding to DNA, and PARP1 gene deletion or inhibition suppresses TNFα-induced release of MMP-9 by microglia and toxicity to co-cultured neurons (53). As illustrated in Figure 2, TNFα activation of PARP1 involves Ca^2+^ influx, stimulation of phosphatidylcholine-specific phospholipase C, and activation of the MEK1/2-ERK1/2 pathway (54), leading to phosphorylation of PARP1 by ERK2 (55). This mechanism does not require DNA damage, which can independently activate PARP *via* stimulation of ERK1/2. One way PARP activity might enable NF-κB binding to DNA is by competing with sirtuin 1 for NAD^+^ substrate. When sirtuin 1 deacetylates the p65 subunit of NF-κB, it is not able to bind to DNA. With excessive activity of PARP sufficient to decrease NAD^+^, NF-κB binding to DNA is increased. This effect is reversed by preventing the decrease in NAD^+^ and is replicated by inhibition of sirtuin 1 (56). Thus, enzymatic activity of PARP1 plays a key role in the activation of NF-κB binding to DNA.

*In vivo*, several pieces of evidence are consistent with PARP inhibitors acting directly to modify the delayed neuroinflammatory response in models of traumatic brain injury (TBI) or ischemia. Rats treated with the PARP inhibitor INO-1001 starting 1 day after TBI exhibited reduced activation of microglia (57), and mice treated with PJ34 starting 1 day after TBI had less neuronal loss in cortex and thalamus and improved sensorimotor function at 3 weeks of recovery (46). Likewise, veliparib decreased expression of pro-inflammatory cytokines and Iba-1 in rats and Iba-1 expression in pigs, even when administration was delayed until 24 h after TBI (58). In a model of global ischemia, administration of PJ34 at 8 h of reperfusion reduced microglial activation while increasing neuronal survival at 7 days (59), and a further delay in treatment until 2 days still resulted in suppressed microglial activation and astrogliosis and increased neuronal density and bromodeoxyuridine staining 8 weeks later (60). With regard to transient MCAO, pre-treatment with PARP inhibitor 3-aminobenzamide (3-AB) decreased MMP-9 activity, neutrophil infiltration, and infarct volume in rats (61), and PJ34 reduced the induction of iNOS and ICAM-1 and decreased infarct volume at 72 h in mice (62). Minocycline is an inhibitor of PARP1 at nanomolar concentrations (63), and this inhibition may be one mechanism by which minocycline reduces post-ischemic neuroinflammation. Finally, a clinical study showed that treating blood samples from stroke patients *ex vivo* with a PARP inhibitor increased the fraction of T-regulatory cells, which are thought to play a role in the brain repair stage after stroke (64).

Therefore, PARP inhibitors are likely to be multipotent in stroke by (1) blocking programmed neuronal necrosis in a large portion of neurons, (2) attenuating the early pro-inflammation response that is thought to be accelerated by reperfusion, and (3) protecting the endothelium and limiting hemorrhagic transformation that is thought to be augmented during aging. This broad spectrum action may be superior to that of drugs such as nerinetide (Tat-NR2B9c), which primarily targets excitotoxicity (18, 65, 66) and failed in the recent Phase III ESCAPE-NA1 trial (67) of ischemic stroke reperfusion therapy.

## Evidence for A Role of Parthanatos in Focal Ischemic Stroke Based on Molecular Interventions in Male Mice

**Figure 3:**
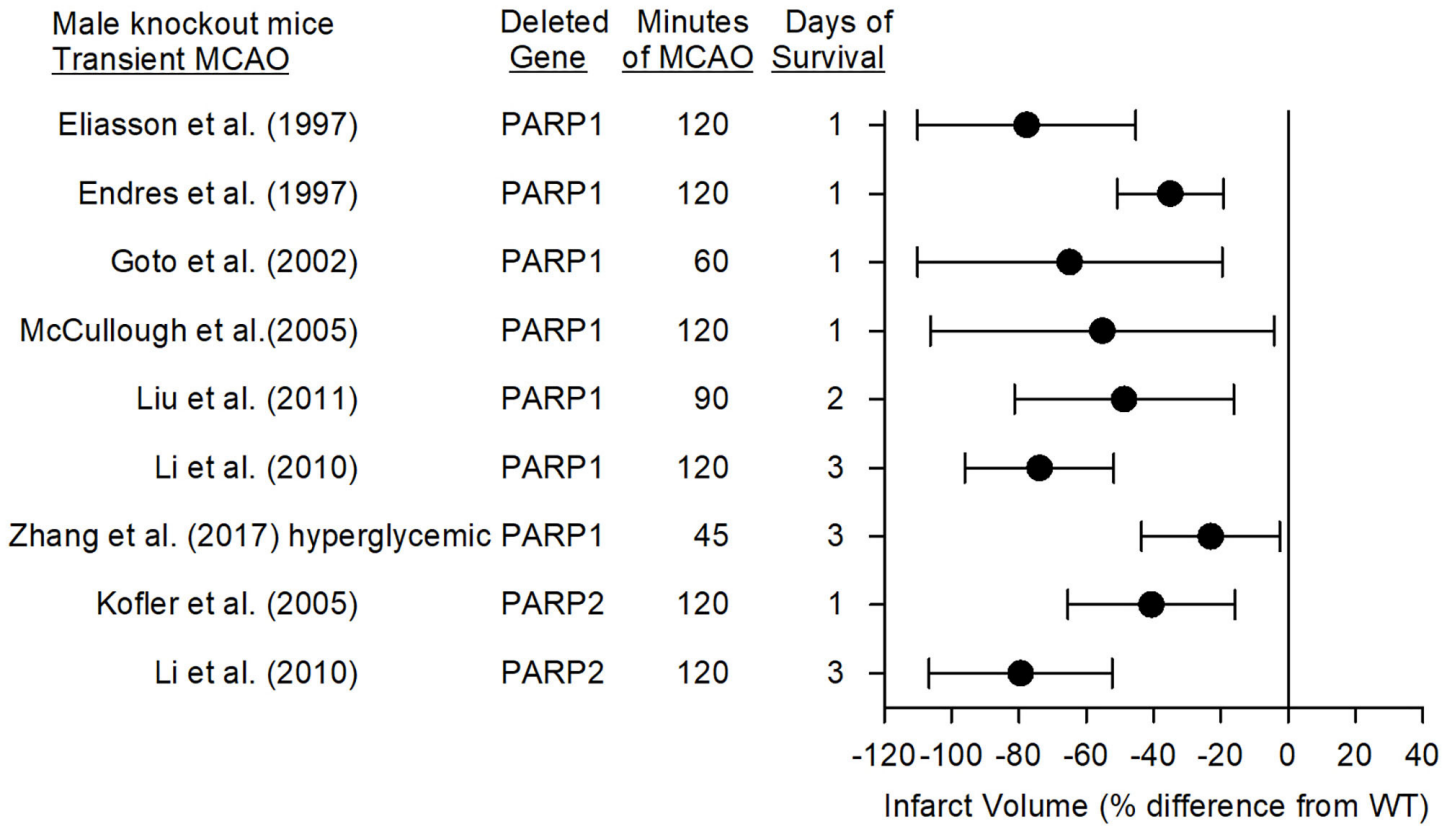
Effect sizes and 95% confidence intervals on infarct volume after transient middle cerebral artery occlusion (MCAO) in male PARP1^−/−^ mice and male PARP2^−/−^ mice, relative to their respective wild-type controls, were derived from mean and SD values and sample sizes extracted from publications by Eliasson et al. (27), Endres et al. (68), Goto et al. (69), McCullough et al. (70), Liu et al. (71), Li et al. (72), Zhang et al. (73), and Kofler et al. (74). These reports from three different laboratories showed significant reductions in infarct volume.

Eight studies have used PARP1-null mice (PARP1^−/−^) and PARP2-null mice (PARP2^−/−^) to investigate the role of PARPs in stroke with models of MCAO. Consistent reductions in infarct volume were seen among these studies emanating from three laboratories. The mean percent difference in infarct volume from the corresponding wild-type (WT) mice and the 95% confidence intervals for these studies with different MCAO durations and different survival times are displayed in Figure 3 for male mice (female mice are discussed in a later section).

### Role of PARP1

In 1997, Eliasson et al. (27) reported that male PARP1^−/−^ mice subjected to 2 h of MCAO did not display the increase in PAR polymer formation seen in WT mice, and that their infarct volume was 80% smaller. PARP heterozygotes displayed a 65% reduction in infarct volume. In the same year, Endres et al. (68, 75) reported that male nNOS^−/−^, PARP1^−/−^, or WT mice pre-treated with 3-AB had decreased infarct volume associated with less PAR polymer formation. PARP is activated by DNA strand breaks, which can be detected in rat striatum at 2 h and in cortex at 4 h after a 2-h MCAO insult (76). Whereas, both the NMDA antagonist MK-801 and the PARP inhibitor 3,4-dihydro-5-[4-(1-piperidinyl)butoxy]-1(2H)-isoquinolinone (DPQ) reduced infarct volume, only MK-801 decreased DNA strand breaks, thereby indicating that NMDA antagonists act upstream and PARP inhibitors act downstream of DNA strand breaks in protecting the brain. Staining of human autopsy tissue from patients who died of stroke revealed increased expression of PARP and PAR polymers in necrotic neurons within and adjacent to the infarct, supporting a role for parthanatos in human stroke (77).

Using MRI, Goto et al. (69) demonstrated that PARP1 contributes to edema and to expansion of infarction over 3 days. At 1 h of MCAO, male PARP1^−/−^ mice had equivalent decreases in the apparent diffusion constant (ADC) and equivalent volumes of low ADC, indicating a similar volume of initial anoxic depolarization. However, by 1 day of reperfusion, the volume of tissue with low ADC was greater in WT than in PARP1^−/−^ mice. T2-weighted determination of infarct volume expanded from 1 through 3 days of reperfusion, whereas the lower infarct volume seen at 1 day in PARP1^−/−^ mice failed to further expand by 3 days, consistent with a role of PARP1 in both neuronal cell death and delayed neuroinflammation.

Because release of AIF from the mitochondria depends on signaling by a large PAR polymer (34, 35), the effect of manipulating PAR polymer availability was tested in mice that overexpressed the PAR degradation enzyme PARG or in mice with heterozygous knockdown of PARG. PARG overexpression decreased infarct volume, whereas PARG knockdown increased infarct volume (35). Furthermore, Iduna is a PAR-dependent ubiquitin E3 ligase (78) whose overexpression reduces PAR polymer availability and attenuates infarct volume after transient MCAO (79). Collectively, these data are consistent with large PAR polymers acting as a death signal to mitochondria in focal ischemia.

The time course of PAR formation and AIF translocation to the nucleus has been examined with different durations of MCAO (80). After 1-h MCAO in male mice, the increase in PAR peaked at 20 min of reperfusion and remained elevated through 24 h. AIF accumulation in the nucleus increased progressively from 20 min to 6 h and from 6 h to 24 h of reperfusion after 1 h of MCAO. Since AIF remains trapped in the nucleus, its accumulation is best related to the time-integral of elevated PAR. In contrast to 1-h MCAO, the increase in nuclear AIF after 30 min of MCAO was delayed beyond 6 h of reperfusion, consistent with a delay of cell death signaling after shorter ischemic durations. The increase in nuclear AIF was delayed after permanent MCAO compared to 1 h of transient MCAO. Thus, reperfusion itself is capable of accelerating AIF nuclear translocation, presumably because a burst of oxidative stress at reperfusion damages nucleic acids. These observations emphasize the need to administer a PARP inhibitor soon after the onset of reperfusion for optimal efficacy. Further, consistent with a role for nNOS in contributing to PARP activation, nNOS^−/−^ mice or administration of an nNOS inhibitor to WT mice reduced formation of PAR polymers and AIF nuclear translocation after transient MCAO.

Parthanatos is associated with large-scale DNA breakdown. Based on work with *C. elegans* homologs of endonuclease G, it had been assumed that mammalian endonuclease G was responsible for executing AIF-dependent DNA degradation. However, infarct volume of endonuclease G^−/−^ mice was equivalent to that of their WT counterparts (81). Moreover, infusion of the PARP inhibitor DR2313 throughout MCAO and early reperfusion decreased infarct volume to a similar extent in WT and endonuclease G^−/−^ male mice, thereby indicating that endonuclease G is not essential for executing parthanatos. Later, it was discovered that MIF serves as an endonuclease in parthanatos when it is complexed to AIF (36). The binding of AIF to MIF in the cytosol promotes uptake of the AIF/MIF complex into the nucleus and activation of MIF's endonuclease activity. Knocking out the MIF gene, altering its binding site to AIF, or altering its endonuclease activity site all were found to markedly reduce infarct volume at 1 and 7 days after 45 min of MCAO in male mice (36).

Because ischemia is associated with acidosis, Zhang et al. (73) explored the role of acidosis in parthanatos. In murine primary neuronal culture, acidosis augmented chemically induced PAR polymer formation, AIF nuclear translocation, and cell death. The augmented AIF translocation and cell death were attenuated by acid-sensitive ion channel 1a and PARP inhibitors. *In vivo*, acute hyperglycemia and its associated severe acidosis are known to augment infarction. Acute hyperglycemia during transient MCAO was found to enhance intraischemic acidosis and augment PAR polymer formation and AIF nuclear translocation, which were attenuated by administration of a PARP inhibitor. Hyperglycemic PARP1^−/−^ mice had a reduction in infarct volume, indicating that parthanatos still plays a role in acidosis-augmented injury.

### Role of PARP2

Kofler et al. (74) reported that infarct volume in PARP2^−/−^ male mice was reduced by 41% compared to that in WT mice at 1 day of recovery, whereas a later study by Li et al. (72) showed a 79% reduction at 3 days of recovery. The latter reduction was comparable to the 74% reduction obtained in PARP1^−/−^ male mice at 3 days after MCAO in the same study. Both knockouts had less PAR formation and AIF nuclear translocation than did WT mice. These reductions were not attributed to differences in cerebral arterial anatomy, MCA distribution volume-at-risk, or regional cerebral blood flow as measured with iodoantipyrine autoradiography. The large protection seen in PARP2^−/−^ mice was unexpected because PARP2 is less abundant than PARP1, suggesting a complex interaction between PARP1 and PARP2 in the context of stroke. PARP inhibitors examined thus far do not exhibit a strong selectivity for PARP1 over PARP2 (82) and hence do not distinguish the role of PARP1 and PARP2 in stroke.

## Efficacy of PARP Inhibitors for Focal Ischemic Stroke in Male Rodents

### Transient Focal Ischemia in Male Mice and Rats

**Figure 4:**
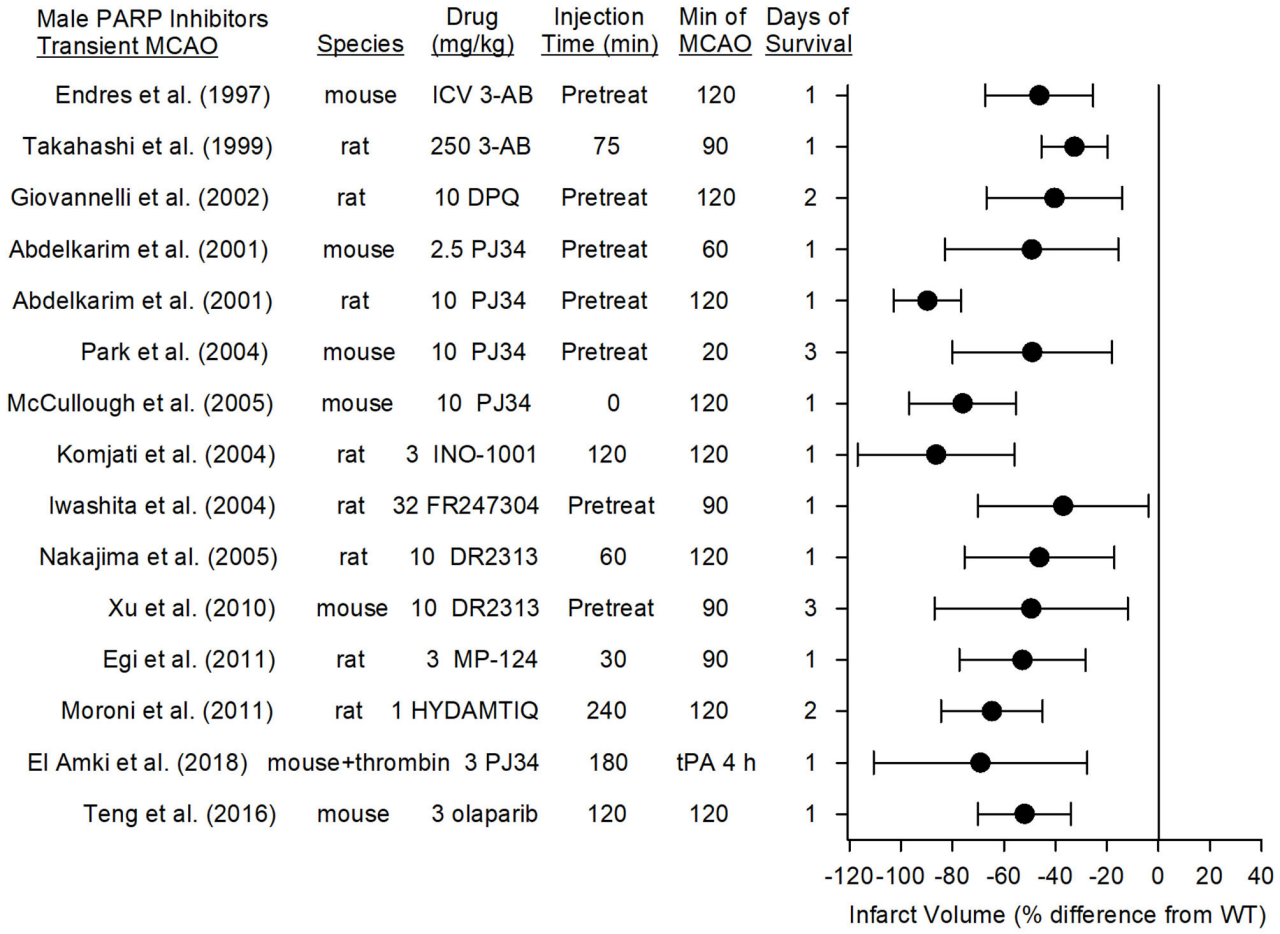
Effect sizes and 95% confidence intervals of PARP inhibitor treatment on infarct volume after transient middle cerebral artery occlusion (MCAO) in male mice and male rats, relative to their respective controls, were derived from mean and SD values and sample sizes extracted from publications by Endres et al. (68), Takahashi and Greenberg (83), Giovannelli et al. (76), Abdelkarim et al. (84), Park et al. (62), McCullough et al. (70), Komjati et al. (85), Iwashita et al. (86), Nakajima et al. (87), Xu et al. (81), Egi et al. (88), Moroni et al. (89), El Amki et al. (90), and Teng et al. (91). These reports from 12 different laboratories using 9 distinct inhibitors showed a significant reduction in infarct volume.

Figure 4 summarizes the effects on infarct volume reported with nine distinct PARP inhibitors in male mice and rats subjected to transient focal ischemia. In some studies, the drugs were administered before inducing the stroke to ensure delivery to the ischemic region, whereas in others, administration was delayed for various times after the onset of stroke or after reperfusion to assess the therapeutic time window. Based on 15 studies carried out between 1997 and 2018 in 13 independent laboratories, PARP inhibition significantly reduced infarct volume from 33 to 86%.

### First Generation PARP Inhibitors

3-AB and DPQ are prototypical PARP inhibitors used for *in vitro* biochemical studies. Early *in vivo* work showed that male mice pre-treated intraventricularly with 3-AB had a 46% decrease in infarct volume associated with less PAR polymer formation (68, 75), and rats pre-treated intraperitoneally with 10 mg/kg DPQ exhibited a 40% reduction in infarct volume (76). However, the relatively high doses required for *in vivo* experiments raise the possibility off-target effects.

### Second Generation PARP Inhibitors

The second generation PJ34 was developed to be a more potent and specific PARP inhibitor than 3-AB and DPQ. Abdelkarim et al. (84) found that pre-treatment with PJ34 decreased infarct volume by 49% in mice subjected to 1-h MCAO and by 90% in rats subjected to 2-h MCAO. Importantly, delaying PJ34 administration until just prior to reperfusion in the rat still reduced infarct volume by 74%. PJ34 was also shown to reduce AIF nuclear translocation (92). Early administration of other specific PARP inhibitors, MP-124 and FR247304, reduced infarct volume by 53 and 37%, respectively, in male rats (86, 88). Interestingly, the potent PARP inhibitor INO-1001 reduced amyloid precursor protein accumulation and AIF translocation in white matter tracts and reduced infarct volume by 86% when administered at reperfusion after 2 h of MCAO in rats (85). Likewise, the PARP inhibitor DR2313 reduced infarct volume by 46% when administration was delayed by 2 h (87) and HTDAMTIQ reduced infarct volume by 65% when delayed by 4 h in rats (89). Thus, several studies using different PARP inhibitors demonstrate neuroprotection in reperfusion models of MCAO. Moreover, in a different reperfusion model in which thrombin was injected directly into the MCA of male mice, administration of rt-PA at 3 h only modestly increased laser Doppler flow (LDF). Administration of PJ34 after the onset of ischemia and again at 3 h substantially improved recovery of LDF, decreased tissue hemoglobin content, decreased infarct volume, and reduced sensorimotor deficits (90). These data suggest that PARP inhibition does not have an adverse interaction with tissue plasminogen activator in enabling neuroprotection.

### Third Generation PARP Inhibitors

Several third generation PARP inhibitors have been designed for their DNA trapping ability for use in human oncology trials, including olaparib, rucaparib, veliparib, talazoparib, niraparib, E7016, and CEP-9722 (93). Of these, olaparib has been tested with MCAO. In randomized groups of male ICR mice that underwent 2-h MCAO, olaparib was injected intraperitoneally at doses of 1, 3, 5, 10, or 25 mg/kg at reperfusion. Doses of 3 and 5 mg/kg, but not higher doses, reduced infarct volume by 52%, attenuated IgG extravasation, and improved ability to use four paws when hanging from a bar (91). In comparisons of the various third generation inhibitors, veliparab had particularly good brain penetration. In rhesus monkeys administered 5 mg/kg veliparib orally, penetration into the cerebrospinal fluid was 57% of the plasma concentration (94). In rats bearing glioma tumors that were treated with 50 mg/kg/d veliparib, the brain concentration (0.72 μg/g excluding glioma) was 77% of the plasma concentration (1.36 μg/g) (95). Veliparib is being tested as a repurposed drug in a multicenter, controlled, randomized pre-clinical trial as part of the Stroke Pre-clinical Assessment Network (https://spannetwork.org/).

### Therapeutic Window of PARP Inhibitors in Transient MCAO

Several studies have directly assessed the effect of variable treatment delays of PARP inhibitors. With 2 h of MCAO in male rats, administration of the PARP inhibitor INO-1001 at 2, 4, or 6 h of MCAO (0, 2, or 4 h of reperfusion) reduced infarct volume by 86, 55, and 27%, respectively when compared to vehicle treatment (85). Similar results were obtained with the PARP inhibitor DR2313, which reduced infarct volume by 62, 47, and 44% when administered to male rats 5 min before 1-h MCAO and 2 and 4 h after MCAO, respectively (87). Likewise, a distinct PARP inhibitor, HYDAMTIQ, administered to male rats 4 h after transient MCAO (2-h duration) reduced infarct volume by 65% at 2 days and 55% at 7 days of recovery (89). Thus, the therapeutic window appears to be in the range of 4–6 h in rats subjected to transient MCAO.

### Permanent MCAO

**Figure 5:**
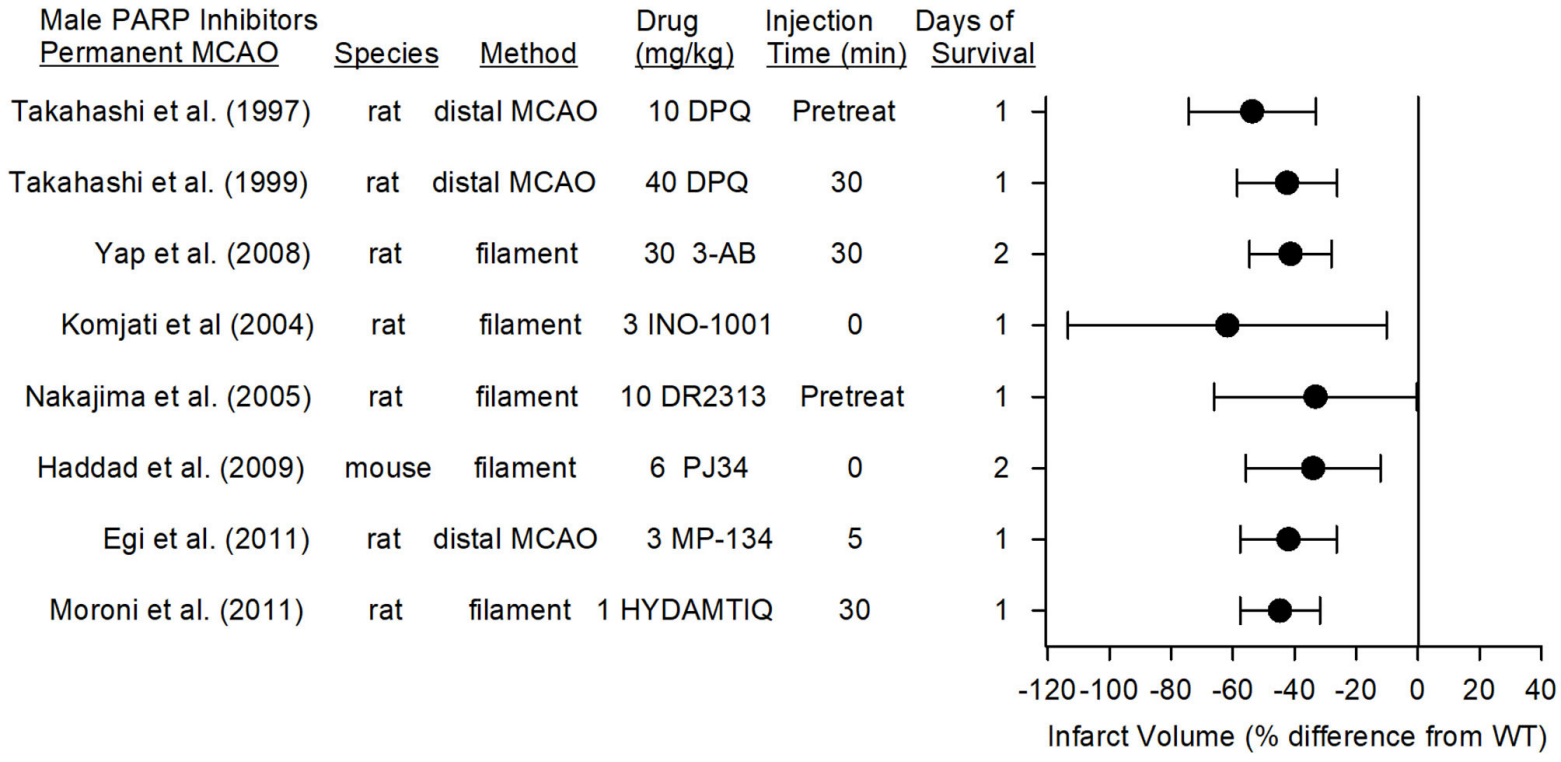
Effect sizes and 95% confidence intervals of PARP inhibitor treatment on infarct volume after permanent middle cerebral artery occlusion (MCAO) in male mice and male rats, relative to their respective controls, were derived from mean and SD values and sample sizes extracted from publications by Takahashi et al. (96), Takahashi et al. (97), Yap et al. (98), Komjati et al. (85), Nakajima et al. (87), Haddad et al. (99), Egi et al. (88), and Moroni et al. (89). These reports from six laboratories using 7 distinct inhibitors showed a significant reduction in infarct volume.

PARP inhibitors also are capable of reducing infarct volume in the distal MCAO model of permanent focal ischemia, which produces primarily a cortical lesion, and with permanent placement of the filament in the internal carotid artery at the MCA root, which causes a larger infarction that includes striatum and cerebral cortex. In eight studies from seven different laboratories, the reduction in infarct volume with seven different PARP inhibitors ranged from 34 to 62% (Figure 5). The range and average effect size of all of the studies was somewhat less than the range and average effect size seen in the transient MCAO models.

In the rat model of permanent distal MCAO plus temporary bilateral carotid occlusion, the PARP inhibitor DPQ reduced infarct volume by 54% when administered as a pre-treatment (96) and by 40% when administered at 30 min after MCAO (97). Likewise, MP-124 administered at 5 min of MCAO reduced infarct volume by 42% in the same model (88).

In the rat permanent filament model, administration of DR2313 as a pre-treatment reduced infarct volume by 33% (87), and administration of INO-1001 at the onset of MCAO reduced infarct volume by 62% (85). 3-AB and HYDAMTIQ, when administered at 30 min of MCAO, reduced infarct volume by 50% (98) and 45% (89), respectively, compared to that of vehicle-treated animals. In the mouse permanent filament model, PJ34 administered at the onset of stroke decreased MMP activity, hemorrhagic transformation, and infarct volume (34% reduction) (99). When recombinant tissue plasminogen activator was given at 6 h of permanent ischemia, the degradation of tight junction proteins, the augmented tissue hemoglobin content, and the augmented sensorimotor deficits induced by recombinant tissue plasminogen activator were ameliorated by PJ34 treatment (100), implying that PARP inhibitors may be beneficial in those without successful recanalization.

The therapeutic window was examined in the rat permanent filament model with INO-1001. This PARP inhibitor, which reduced infarct volume by 62% when administered at the onset of permanent MCAO, remained effective in reducing infarct volume by 59% when administration was delayed by 2 h (85). When the delay was extended to 4 h, the reduction was 40%, but not statistically significant, most likely because this group was underpowered (*n* = 6). Thus, multiple PARP inhibitors are effective in models of permanent MCAO, and the therapeutic window is ~2 h.

## Role of Parthanatos in Female Animals With Focal Ischemic Stroke

### Female Mice

Young female mice and rats generally have smaller infarcts than their male counterparts. Some of this sex difference is attributable to estrogen because ovariectomy increases infarct size whereas estrogen replacement reduces infarct size (101, 102). It has often been difficult to show further reductions in infarct volume beyond that afforded by endogenous sex hormones in young healthy female rodents. The preponderance of studies that have investigated the use of PARP inhibitors for MCAO have used male animals. A sex difference in PARP-dependent ischemic injury was first noted in neonatal mice exposed to hypoxia-ischemia. In that study, PARP1^−/−^ males were protected whereas PARP1^−/−^ females were not (103). McCullough et al. (70) investigated sex differences in cell death mechanisms of mature brain in detail in 3-month-old mice. They first reported that female PARP^−/−^ mice or WT mice administered PJ34 do not exhibit decreases in infarct volume after transient MCAO. To the contrary, infarct volume measured at 24 h was significantly increased, and the percent increase was large on account of the small infarct size in the WT females. The female WT mice exhibited an increase in PAR and nuclear AIF, although the increase in PAR was less than that seen in males (104). They went on to show that canonical apoptosis played a larger role in injury from MCAO in young female mice (105), an effect that was augmented in female PARP^−/−^ mice (71). The augmented stroke injury in female PARP^−/−^ mice was associated with augmented decreases in NAD^+^ (106), which may reduce the protective effect of NAD^+^-dependent SIRT deacetylase activity in limiting NF-κB transcription (56).

Because estrogen is known to account for the smaller infarct volume in WT females than in WT males, Liu et al. (71) also studied young female mice 2 weeks after ovariectomy and found an infarct size comparable to that in WT males. Ovariectomized PARP^−/−^ mice did not display a decrease in infarct volume compared to that of ovariectomized WT mice. Infarct volume could still be reduced with a caspase inhibitor after ovariectomy in both WT and PARP^−/−^ mice. When survival was extended to 48 h and permanent MCAO was induced in ovariectomized PARP^−/−^ mice, protection was not seen (71). Thus, elevated estrogen levels in young female mice did not completely account for the observed sex differences in the role of PARP and classical apoptosis. However, three limitations should be noted with regard to clinical translation. (1) Though lack of PARP1 was not protective in young ovariectomized rats, a PARP inhibitor was not tested to distinguish a possible effect of lifelong PARP1 absence in young ovariectomized WT mice. (2) Although gross neurologic deficits were reported at 24 h after transient MCAO, long-term behavioral outcomes and neuroinflammation have not been reported with a PARP inhibitor or gene deletion in intact or ovariectomized female mice. (3) Acute or long-term outcomes have not been reported with a PARP inhibitor or gene deletion in middle-aged or older female mice, when protection by estrogen is lost.

### Female Rats

**Figure 6:**
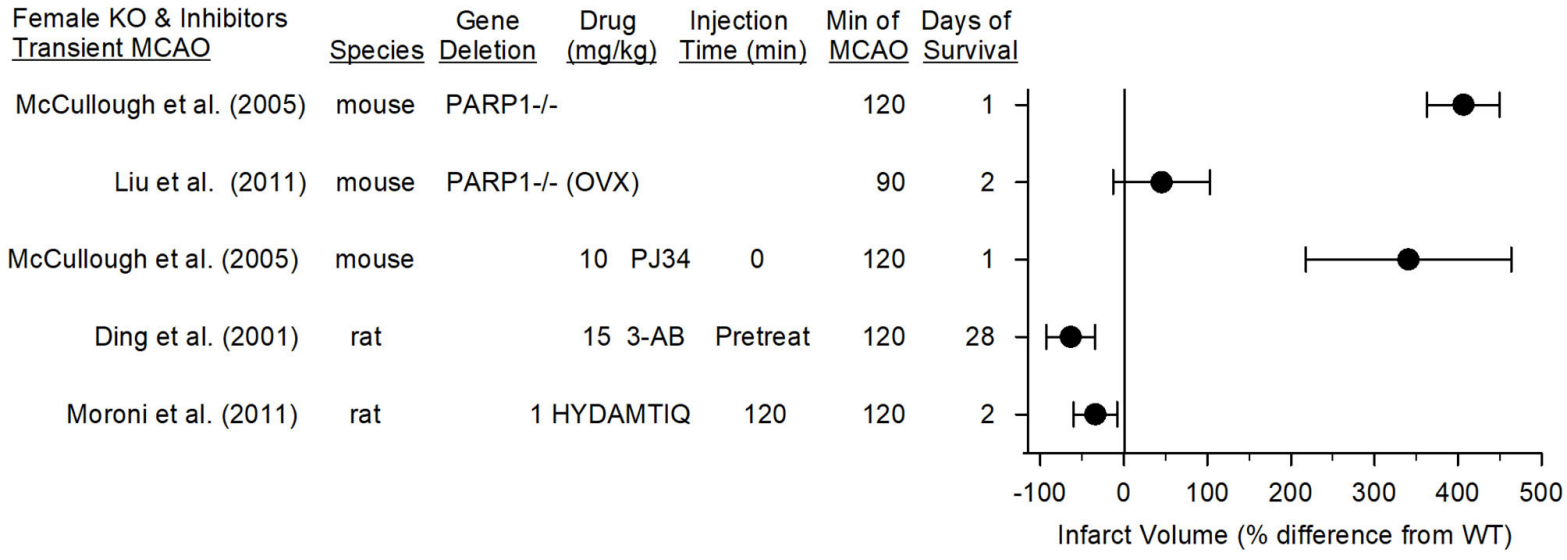
Effect sizes and 95% confidence intervals on infarct volume after transient middle cerebral artery occlusion (MCAO) in female PARP1^−/−^ mice and female mice treated with a PARP inhibitor, relative to their respective controls, were derived from mean and SD values and sample sizes extracted from publications by McCullough et al. (70), Liu et al. (71), Ding et al. (107), and Moroni et al. (89). On a percentage basis, the large increase in infarct volume in PARP1^−/−^ mice is influenced by the small infarcts in the wild-type (WT) female mice. Ovariectimized (OVX) WT mice had larger infarcts than non-OVX WT mice, and the OVX PARP^−/−^ mice did not have a significant increase in infarct volume compared to OVX WT mice.

In contrast to this work in female PARP1^−/−^ mice, two laboratories have reported positive effects of PARP inhibitors in young female rats. Moroni et al. (89) found that administering 1 mg/kg of the PARP inhibitor HYDAMTIQ at reperfusion after 2 h of MCAO in female rats decreased infarct volume at 2 days by 34% (Figure 6). This reduction was <65% reduction seen in male mice with the same protocol. However, a higher dose (10 mg/kg) of a more hydrophobic form of this inhibitor (DAMTIQ) did produce similar infarct volume reductions in females and males.

Pre-treatment of female rats with 3-AB before 2-h MCAO reduced infarct volume and improved motor performance on the foot-fault, parallel bar, rope climbing, and ladder climbing tests over 28 days of recovery (107). Thus, it is unclear if the observed effect of PARP1 gene deletion in young female mice can be extended to use of a PARP inhibitor in other species. Of note, Tat-NR2Bc, which interrupts the membrane association of nNOS with the NR2B subunit and PSD95 (18) and decreases superoxide production by NADPH oxidase (20), is equally effective in markedly reducing infarction in male and female rats when given 1 h after permanent occlusion of a distal pial artery (108). Because this PSD95 coupling plays a critical role in excitotoxic damage that leads to peroxynitrite-induced DNA damage and PARP activation, these data also suggest that parthanatos likely plays an important role in female rats.

One final piece of evidence indicates that sex is less important in human neurons exposed to NMDA or OGD. In cultured human neurons, those with a female genotype and those with a male genotype exhibited similar increases in PAR, nuclear AIF, and cell death, and both were salvaged equivalently by treatment with an nNOS inhibitor or several third generation PARP inhibitors (29). Because sex can influence the inflammatory response, more work is needed to determine possible sex differences in how PARP modifies inflammation and evolution of the infarction process. In any case, current evidence does not make clear whether sex differences in parthanatos seen in mouse cultured neurons (109) or neonatal (103) or 3-month-old mice (70) would persist in aged animals.

## Scientific Rigor for Clinical Translation of PARP Inhibitors

### Randomization and Treatment Concealment

Most of the stroke studies carried out with PARP inhibitors and PARP^−/−^ mice were performed before 2010, and many did not formally comment on randomization and blinding of treatment groups. Studies low in quality criteria of scientific rigor are thought to overestimate the true effect size. On the other hand, a large number of studies from multiple labs with many different PARP inhibitors consistently reported reductions in infarct volume with *n* = 6–20 per study. Although we did not conduct a formal meta-analysis, the consistent infarct reductions with a large effect size and reasonable sample sizes mitigates some of the quality concerns of the early studies.

### PARP Inhibitor Experiments With Negative Findings

Publication bias of reporting only positive findings is always a concern, and whether negative findings have gone unreported, especially for young female animals, is unknown. In our literature survey, we found two experiments with negative results embedded in papers with otherwise positive results. Takahashi and Greenberg (83) reported that whereas 3-AB pre-treatment in rats reduced infarct volume, treatment at 15 min after reperfusion failed to do so (*n* = 6). Abdelkarim et al. (84) reported that whereas pre-treatment with PJ34 protected rats from 4 h of permanent MCAO, pre-treatment failed to protect from 24 h of permanent MCAO with the filament technique (*n* = 8–9). The latter effect could be explained by an inadequate plasma half-life when ischemia extends beyond 4 h. Positive effects of INO-1001 in permanent MCAO utilized multiple injections over 24 h (85). Thus, the negative findings in these studies may be explained by an ineffective dosing regimen and possibly by small sample sizes.

### Large-Animal Models

Some early candidate treatments, such as tirilazad (110), monoclonal leukocyte antibody (111), and a hemoglobin-based oxygen carrier (112), that showed positive results in rodent models were not positive in large-animal stroke models or in human trials. Many reasons might explain why early human stroke trials with acute neuroprotectants failed, including rapid movement of a candidate drug from rodent models to human trials without testing in a large animal that has a brain more complex than a rodent's. Although PARP inhibitors have not been tested in large-animal models of stroke, INO-1001 was shown to protect neurons from spinal cord ischemia in pigs (113), and velaparib was shown to reduce microglial activation after TBI in pigs (58). These studies provide some assurance that PARP-dependent mechanisms of neuronal cell death and inflammation are operative in higher species. Nevertheless, the field would benefit from confirmation that PARP inhibitors are beneficial in a large-animal model of focal stroke in both males and females.

### Neurobehavior Outcome

Many of the early studies were limited by the short survival time and reliance on only infarct volume as the primary outcome measurement. Translational stroke studies now place greater emphasis on neurobehavior outcomes. Whereas, a few studies with PARP inhibitors included acute sensorimotor deficits in the study design (90, 100), only two presented long-term neurobehavior outcomes. Nevertheless, these studies demonstrated persistent positive effects of PARP inhibitors on sensorimotor tests at 28 days in female rats (107) and 90 days in male rats (89).

### Co-morbidities

One important factor that is thought to contribute to the poor track record for clinical translation of stroke therapies is that most animal stroke studies are performed in young healthy animals. This is also true for all of the aforementioned studies with PARP inhibitors. Therefore, future studies should consider testing these inhibitors in aged animals and in those with chronic disorders seen in the human stroke population, such as hypertension, diabetes, obesity, and high-salt diet.

## Conclusions

Multiple forms of programmed cell death, including parthanatos, RIP1/RIP3-dependent necroptosis, and classical apoptosis, are operative after the onset of ischemic stroke. On the basis of infarct volume reduction, parthanatos is a predominate form, at least in male animals. In young female animals in whom sex hormones provide some protection compared to males, caspase-dependent apoptosis plays a more significant role than in young male animals. Nevertheless, a limited set of studies with long-term behavior outcomes support a benefit of PARP inhibitor treatment in male and female animals. In addition to blocking parthanatos, some of the benefit of PARP inhibition is likely derived from attenuation of NF-κB transcription in microglia, astrocytes, and peripheral immune cells, although these anti-inflammatory effects have not been as well-studied in the context of stroke as they have in the context of TBI. Support for possible clinical translation of PARP inhibitors in stroke is favored by (1) positive effects on infarct volume reported by 13 independent laboratories with 9 different drugs, (2) efficacy in both transient and permanent focal ischemia, (3) a therapeutic window in the range of 4–6 h for infarct volume reduction, and (4) availability of PARP inhibitors with a good safety profile with month-long administration in human oncology trials. However, before clinical translation some limitations in the current literature would need to be addressed, including (1) resolving possible species differences in efficacy in young female animals, (2) performing additional pre-clinical studies with a high level of scientific rigor quality, (3) assessing the therapeutic window for neurobehavior outcome, which may differ from the therapeutic window for infarct volume reduction, (4) assessing efficacy in aged animals and those with cardiovascular risk factors, and (5) possibly testing in a large animal species with gyrencephalic brain.

## Author Contributions

RK analyzed the data in the literature and drafted the manuscript. VD and TD edited the manuscript. All authors contributed to the article and approved the submitted version.

## Conflict of Interest

TD and VD are inventors of technology discussed in this this publication, which Neuraly, Inc. is in the process of licensing from Johns Hopkins University. TD and VD are founders of Neuraly, and hold shares of stock options as well as equity in, Neuraly, Inc., which is a subsidiary of D&D Pharmatech Inc. This arrangement has been reviewed and approved by the Johns Hopkins University in accordance with its conflict of interest policies. The remaining author declares that the research was conducted in the absence of any commercial or financial relationships that could be construed as a potential conflict of interest.

## References

[1] Berkhemer OA Fransen PS Beumer D van den Berg LA Lingsma HF Yoo AJ. A randomized trial of intraarterial treatment for acute ischemic stroke. N Engl J Med. (2015) 372:11–20. 10.1056/NEJMoa1411587 25517348

[2] Campbell BC Mitchell PJ Kleinig TJ Dewey HM Churilov L Yassi N. Endovascular therapy for ischemic stroke with perfusion-imaging selection. N Engl J Med. (2015) 372:1009–18. 10.1056/NEJMoa1414792 25671797

[3] Goyal M Demchuk AM Menon BK Eesa M Rempel JL Thornton J. Randomized assessment of rapid endovascular treatment of ischemic stroke. N Engl J Med. (2015) 372:1019–30. 10.1056/NEJMoa1414905 25671798

[4] Jovin TG Chamorro A Cobo E de Miquel MA Molina CA Rovira A. Thrombectomy within 8 hours after symptom onset in ischemic stroke. N Engl J Med. (2015) 372:2296–306. 10.1056/NEJMoa1503780 25882510

[5] Saver JL Goyal M Bonafe A Diener HC Levy EI Pereira VM. Stent-retriever thrombectomy after intravenous t-PA vs. t-PA alone in stroke. N Engl J Med. (2015) 372:2285–95. 10.1056/NEJMoa1415061 25882376

[6] Albers GW Marks MP Kemp S Christensen S Tsai JP Ortega-Gutierrez S. Thrombectomy for stroke at 6 to 16 hours with selection by perfusion imaging. N Engl J Med. (2018) 378:708–18. 10.1056/NEJMoa1713973 29364767 PMC6590673

[7] Nogueira RG Jadhav AP Haussen DC Bonafe A Budzik RF Bhuva P. Thrombectomy 6 to 24 hours after stroke with a mismatch between deficit and infarct. N Engl J Med. (2018) 378:11–21. 10.1056/NEJMoa1706442 29129157

[8] Shi L Rocha M Leak RK Zhao J Bhatia TN Mu H. A new era for stroke therapy: integrating neurovascular protection with optimal reperfusion. J Cereb Blood Flow Metab. (2018) 38:2073–91. 10.1177/0271678X18798162 30191760 PMC6282224

[9] Bosetti F Koenig JI Ayata C Back SA Becker K Broderick JP. Translational stroke research: vision and opportunities. Stroke. (2017) 48:2632–7. 10.1161/STROKEAHA.117.017112 28751554 PMC5599159

[10] Bix GJ Fraser JF Mack WJ Carmichael ST Perez-Pinzon M Offner H. Uncovering the Rosetta stone: report from the first annual conference on key elements in translating stroke therapeutics from pre-clinical to clinical. Transl Stroke Res. (2018) 9:258–66. 10.1007/s12975-018-0628-9 29633156 PMC5982459

[11] Zhang J Pieper A Snyder SH. Poly(ADP-ribose) synthetase activation: an early indicator of neurotoxic DNA damage. J Neurochem. (1995) 65:1411–4. 7643121 10.1046/j.1471-4159.1995.65031411.x

[12] Lorch S Lightfoot R Ohshima H Virag L Chen Q Hertkorn C. Detection of peroxynitrite-induced protein and DNA modifications. Methods Mol Biol. (2002) 196:247–75. 10.1385/1-59259-274-0:247 12152205

[13] Beckman JS Koppenol WH. Nitric oxide, superoxide, and peroxynitrite: the good, the bad, and ugly. Am J Physiol Cell Physiol. (1996) 271:C1424–37. 8944624 10.1152/ajpcell.1996.271.5.C1424

[14] Eliasson MJ Huang Z Ferrante RJ Sasamata M Molliver ME Snyder SH. Neuronal nitric oxide synthase activation and peroxynitrite formation in ischemic stroke linked to neural damage. J Neurosci. (1999) 19:5910–8. 10.1523/JNEUROSCI.19-14-05910.1999 10407030 PMC6783066

[15] Fukuyama N Takizawa S Ishida H Hoshiai K Shinohara Y Nakazawa H. Peroxynitrite formation in focal cerebral ischemia-reperfusion in rats occurs predominantly in the peri-infarct region. J Cereb Blood Flow Metab. (1998) 18:123–9. 9469153 10.1097/00004647-199802000-00001

[16] Hartings JA Shuttleworth CW Kirov SA Ayata C Hinzman JM Foreman B. The continuum of spreading depolarizations in acute cortical lesion development: examining Leao's legacy. J Cereb Blood Flow Metab. (2017) 37:1571–94. 10.1177/0271678X16654495 27328690 PMC5435288

[17] Zhang LN Hao L Guo YS Wang HY Li LL Liu LZ. Are glutamate transporters neuroprotective or neurodegenerative during cerebral ischemia? J Mol Med (Berl). (2019) 97:281–9. 10.1007/s00109-019-01745-5 30675649

[18] Sattler R Xiong Z Lu WY Hafner M MacDonald JF Tymianski M. Specific coupling of NMDA receptor activation to nitric oxide neurotoxicity by PSD-95 protein. Science. (1999) 284:1845–8. 10364559 10.1126/science.284.5421.1845

[19] Dawson TM Dawson VL. Mitochondrial mechanisms of neuronal cell death: potential therapeutics. Annu Rev Pharmacol Toxicol. (2017) 57:437–54. 10.1146/annurev-pharmtox-010716-105001 28061689 PMC11323062

[20] Chen Y Brennan-Minnella AM Sheth S El-Benna J Swanson RA. Tat-NR2B9c prevents excitotoxic neuronal superoxide production. J Cereb Blood Flow Metab. (2015) 35:739–42. 10.1038/jcbfm.2015.16 25669908 PMC4420863

[21] Minnella AM Zhao JX Jiang X Jakobsen E Lu F Wu L. Excitotoxic superoxide production and neuronal death require both ionotropic and non-ionotropic NMDA receptor signaling. Sci Rep. (2018) 8:17522. 10.1038/s41598-018-35725-5 30504838 PMC6269523

[22] Narasimhan P Fujimura M Noshita N Chan PH. Role of superoxide in poly(ADP-ribose) polymerase upregulation after transient cerebral ischemia. Brain Res Mol Brain Res. (2003) 113:28–36. 10.1016/s0169-328x(03)00062-7 12750003

[23] Benveniste H Hansen AJ Ottosen NS. Determination of brain interstitial concentrations by microdialysis. J Neurochem. (1989) 52:1741–50. 2723633 10.1111/j.1471-4159.1989.tb07252.x

[24] Benveniste H. The excitotoxin hypothesis in relation to cerebral ischemia. Cerebrovasc Brain Metab Rev. (1991) 3:213–45. 1931486

[25] Yang ZJ Carter EL Torbey MT Martin LJ Koehler RC. Sigma receptor ligand 4-phenyl-1-(4-phenylbutyl)-piperidine modulates neuronal nitric oxide synthase/postsynaptic density-95 coupling mechanisms and protects against neonatal ischemic degeneration of striatal neurons. Exp Neurol. (2010) 221:166–74. 10.1016/j.expneurol.2009.10.019 19883643 PMC2812675

[26] Zhang J Dawson VL Dawson TM Snyder SH. Nitric oxide activation of poly(ADP-ribose) synthetase in neurotoxicity. Science. (1994) 263:687–9. 8080500 10.1126/science.8080500

[27] Eliasson MJL Sampei K Mandir AS Hurn PD Traystman RJ Bao J. Poly(ADP-ribose) polymerase gene disruption renders mice resistant to cerebral ischemia. Nature Med. (1997) 3:1089–95. 9334719 10.1038/nm1097-1089

[28] Mandir AS Poitras MF Berliner AR Herring WJ Guastella DB Feldman A. NMDA but not non-NMDA excitotoxicity is mediated by Poly(ADP-ribose) polymerase. J Neurosci. (2000) 20:8005–11. 10.1523/JNEUROSCI.20-21-08005.2000 11050121 PMC6772735

[29] Xu JC Fan J Wang X Eacker SM Kam TI Chen L. Cultured networks of excitatory projection neurons and inhibitory interneurons for studying human cortical neurotoxicity. Sci Transl Med. (2016) 8:333ra48. 10.1126/scitranslmed.aad0623 27053772 PMC5595216

[30] Yu SW Wang H Poitras MF Coombs C Bowers WJ Federoff HJ. Mediation of poly(ADP-ribose) polymerase-1-dependent cell death by apoptosis-inducing factor. Science. (2002) 297:259–63. 10.1126/science.1072221 12114629

[31] Wang H Yu SW Koh DW Lew J Coombs C Bowers W. Apoptosis-inducing factor substitutes for caspase executioners in NMDA-triggered excitotoxic neuronal death. J Neurosci. (2004) 24:10963–73. 10.1523/JNEUROSCI.3461-04.2004 15574746 PMC6730219

[32] Yu SW Wang Y Frydenlund DS Ottersen OP Dawson VL Dawson TM. Outer mitochondrial membrane localization of apoptosis-inducing factor: mechanistic implications for release. ASN Neuro. (2009) 1:e00021. 10.1042/AN20090046 19863494 PMC2784601

[33] Wang Y Kim NS Haince JF Kang HC David KK Andrabi SA. Poly(ADP-ribose) (PAR) binding to apoptosis-inducing factor is critical for PAR polymerase-1-dependent cell death (parthanatos). Sci Signal. (2011) 4(167):ra20. 10.1126/scisignal.2000902 21467298 PMC3086524

[34] Yu SW Andrabi SA Wang H Kim NS Poirier GG Dawson TM. Apoptosis-inducing factor mediates poly(ADP-ribose) (PAR) polymer-induced cell death. Proc Natl Acad Sci USA. (2006) 103:18314–9. 10.1073/pnas.0606528103 17116881 PMC1838748

[35] Andrabi SA Kim NS Yu SW Wang H Koh DW Sasaki M. Poly(ADP-ribose) (PAR) polymer is a death signal. Proc Natl Acad Sci USA. (2006) 103(48):18308-13. 10.1073/pnas.0606526103 17116882 PMC1838747

[36] Wang Y An R Umanah GK Park H Nambiar K Eacker SM. A nuclease that mediates cell death induced by DNA damage and poly(ADP-ribose) polymerase-1. Science. (2016) 354:aad6872. 10.1126/science.aad6872 27846469 PMC5134926

[37] Park H Kam TI Dawson TM Dawson VL. Poly (ADP-ribose) (PAR)-dependent cell death in neurodegenerative diseases. Int Rev Cell Mol Biol. (2020) 353:1–29. 10.1016/bs.ircmb.2019.12.009 32381174

[38] Degterev A Huang Z Boyce M Li Y Jagtap P Mizushima N. Chemical inhibitor of nonapoptotic cell death with therapeutic potential for ischemic brain injury. Nat Chem Biol. (2005) 1:112–9. 10.1038/nchembio711 16408008

[39] Liu C Fang Y. New insights of poly(ADP-ribosylation) in neurodegenerative diseases: a focus on protein phase separation and pathologic aggregation. Biochem Pharmacol. (2019) 167:58–63. 10.1016/j.bcp.2019.04.028 31034795

[40] Mabley JG Jagtap P Perretti M Getting SJ Salzman AL Virag L. Anti-inflammatory effects of a novel, potent inhibitor of poly (ADP-ribose) polymerase. Inflamm Res. (2001) 50:561–9. 10.1007/PL00000234 11766996

[41] Radovits T Seres L Gero D Berger I Szabo C Karck M. Single dose treatment with PARP-inhibitor INO-1001 improves aging-associated cardiac and vascular dysfunction. Exp Gerontol. (2007) 42:676–85. 10.1016/j.exger.2007.01.013 17383839 PMC2684519

[42] Garcia Soriano F Virag L Jagtap P Szabo E Mabley JG Liaudet L. Diabetic endothelial dysfunction: the role of poly(ADP-ribose) polymerase activation. Nat Med. (2001) 7:108–13. 10.1038/83241 11135624

[43] Radovits T Zotkina J Lin LN Bomicke T Arif R Gero D. Poly(ADP-Ribose) polymerase inhibition improves endothelial dysfunction induced by hypochlorite. Exp Biol Med (Maywood). (2007) 232:1204–12. 10.3181/0701-RM-16 17895528

[44] Radovits T Lin LN Zotkina J Gero D Szabo C Karck M. Poly(ADP-ribose) polymerase inhibition improves endothelial dysfunction induced by reactive oxidant hydrogen peroxide *in vitro*. Eur J Pharmacol. (2007) 564:158–66. 10.1016/j.ejphar.2007.02.060 17397824

[45] Bakondi E Bai P Szabo EE Hunyadi J Gergely P Szabo C. Detection of poly(ADP-ribose) polymerase activation in oxidatively stressed cells and tissues using biotinylated NAD substrate. J Histochem Cytochem. (2002) 50:91–8. 10.1177/002215540205000110 11748298

[46] Stoica BA Loane DJ Zhao Z Kabadi SV Hanscom M Byrnes KR. PARP-1 inhibition attenuates neuronal loss, microglia activation and neurological deficits after traumatic brain injury. J Neurotrauma. (2014) 31:758–72. 10.1089/neu.2013.3194 24476502 PMC3967421

[47] Lenzser G Kis B Snipes JA Gaspar T Sandor P Komjati K. Contribution of poly(ADP-ribose) polymerase to postischemic blood-brain barrier damage in rats. J Cereb Blood Flow Metab. (2007) 27:1318–26. 10.1038/sj.jcbfm.9600437 17213862

[48] Mehrabadi AR Korolainen MA Odero G Miller DW Kauppinen TM. Poly(ADP-ribose) polymerase-1 regulates microglia mediated decrease of endothelial tight junction integrity. Neurochem Int. (2017) 108:266–71. 10.1016/j.neuint.2017.04.014 28461173

[49] Zhang Y Zhang X Park TS Gidday JM. Cerebral endothelial cell apoptosis after ischemia-reperfusion: role of PARP activation and AIF translocation. J Cereb Blood Flow Metab. (2005) 25:868–77. 10.1038/sj.jcbfm.9600081 15729291

[50] Xu J Wang H Won SJ Basu J Kapfhamer D Swanson RA. Microglial activation induced by the alarmin S100B is regulated by poly(ADP-ribose) polymerase-1. Glia. (2016) 64:1869–78. 10.1002/glia.23026 27444121 PMC7927976

[51] Raghunatha P Vosoughi A Kauppinen TM Jackson MF. Microglial NMDA receptors drive pro-inflammatory responses *via* PARP-1/TRMP2 signaling. Glia. (2020) 68:1421–34. 10.1002/glia.23790 32036619

[52] Chiarugi A Moskowitz MA. Poly(ADP-ribose) polymerase-1 activity promotes NF-kappaB-driven transcription and microglial activation: implication for neurodegenerative disorders. J Neurochem. (2003) 85:306–17. 10.1046/j.1471-4159.2003.01684.x 12675907

[53] Kauppinen TM Swanson RA. Poly(ADP-ribose) polymerase-1 promotes microglial activation, proliferation, and matrix metalloproteinase-9-mediated neuron death. J Immunol. (2005) 174:2288–96. 10.4049/jimmunol.174.4.2288 15699164

[54] Vuong B Hogan-Cann AD Alano CC Stevenson M Chan WY Anderson CM. NF-kappaB transcriptional activation by TNFalpha requires phospholipase C, extracellular signal-regulated kinase 2 and poly(ADP-ribose) polymerase-1. J Neuroinflammation. (2015) 12:229. 10.1186/s12974-015-0448-8 26637332 PMC4670503

[55] Kauppinen TM Chan WY Suh SW Wiggins AK Huang EJ Swanson RA. Direct phosphorylation and regulation of poly(ADP-ribose) polymerase-1 by extracellular signal-regulated kinases 1/2. Proc Natl Acad Sci USA. (2006) 103:7136–41. 10.1073/pnas.0508606103 16627622 PMC1459030

[56] Kauppinen TM Gan L Swanson RA. Poly(ADP-ribose) polymerase-1-induced NAD(+) depletion promotes nuclear factor-kappaB transcriptional activity by preventing p65 de-acetylation. Biochim Biophys Acta. (2013) 1833:1985–91. 10.1016/j.bbamcr.2013.04.005 23597856 PMC4041949

[57] d'Avila JC Lam TI Bingham D Shi J Won SJ Kauppinen TM. Microglial activation induced by brain trauma is suppressed by post-injury treatment with a PARP inhibitor. J Neuroinflammation. (2012) 9:31. 10.1186/1742-2094-9-31 22335939 PMC3298794

[58] Irvine K Bishop R Won SJ Xu J Hamel K Coppes V. Effects of veliparib on microglial activation and functional outcomes following traumatic brain injury in the rat and pig. J Neurotrauma. (2018) 35:918–29. 10.1089/neu.2017.5044 29285982

[59] Hamby AM Suh SW Kauppinen TM Swanson RA. Use of a poly(ADP-ribose) polymerase inhibitor to suppress inflammation and neuronal death after cerebral ischemia-reperfusion. Stroke. (2007) 38(Suppl. 2):632–6. 10.1161/01.STR.0000250742.61241.79 17261705

[60] Kauppinen TM Suh SW Berman AE Hamby AM Swanson RA. Inhibition of poly(ADP-ribose) polymerase suppresses inflammation and promotes recovery after ischemic injury. J Cereb Blood Flow Metab. (2009) 29:820–9. 10.1038/jcbfm.2009.9 19190653

[61] Koh SH Park Y Song CW Kim JG Kim K Kim J. The effect of PARP inhibitor on ischaemic cell death, its related inflammation and survival signals. Eur J Neurosci. (2004) 20:1461–72. 10.1111/j.1460-9568.2004.03632.x 15355313

[62] Park EM Cho S Frys K Racchumi G Zhou P Anrather J. Interaction between inducible nitric oxide synthase and poly(ADP-ribose) polymerase in focal ischemic brain injury. Stroke. (2004) 35:2896–901. 10.1161/01.STR.0000147042.53659.6c 15514191

[63] Alano CC Ying W Swanson RA. Poly(ADP-ribose) polymerase-1-mediated cell death in astrocytes requires NAD^+^ depletion and mitochondrial permeability transition. J Biol Chem. (2004) 279:18895–902. 10.1074/jbc.M313329200 14960594

[64] Noh MY Lee WM Lee SJ Kim HY Kim SH Kim YS. Regulatory T cells increase after treatment with poly (ADP-ribose) polymerase-1 inhibitor in ischemic stroke patients. Int Immunopharmacol. (2018) 60:104–10. 10.1016/j.intimp.2018.04.043 29709770

[65] Aarts M Liu Y Liu L Besshoh S Arundine M Gurd JW. Treatment of ischemic brain damage by perturbing NMDA receptor- PSD-95 protein interactions. Science. (2002) 298:846–50. 10.1126/science.1072873 12399596

[66] Cook DJ Teves L Tymianski M. A translational paradigm for the preclinical evaluation of the stroke neuroprotectant Tat-NR2B9c in gyrencephalic nonhuman primates. Sci Transl Med. (2012) 4:154ra33. 10.1126/scitranslmed.3003824 23035045

[67] Hill MD Goyal M Menon BK Nogueira RG McTaggart RA Demchuk AM. Efficacy and safety of nerinetide for the treatment of acute ischaemic stroke (ESCAPE-NA1): a multicentre, double-blind, randomised controlled trial. Lancet. (2020) 395:878–87. 10.1016/S0140-6736(20)30258-0 32087818

[68] Endres M Wang ZQ Namura S Waeber C Moskowitz MA. Ischemic brain injury is mediated by the activation of poly(ADP- ribose)polymerase. J Cereb Blood Flow Metab. (1997) 17:1143–51. 9390645 10.1097/00004647-199711000-00002

[69] Goto S Xue R Sugo N Sawada M Poitras M Johns DC. Poly (ADP-ribose) polymerase (PARP-1) impairs early and long-term experimental stroke therapy. Stroke. (2002) 33:1101–6. 10.1161/01.str.0000014203.65693.1e 11935067

[70] McCullough LD Zeng Z Blizzard KK Debchoudhury I Hurn PD. Ischemic nitric oxide and poly (ADP-ribose) polymerase-1 in cerebral ischemia: male toxicity, female protection. J Cereb Blood Flow Metab. (2005) 25:502–12. 10.1038/sj.jcbfm.9600059 15689952

[71] Liu F Lang J Li J Benashski SE Siegel M Xu Y. Sex differences in the response to poly(ADP-ribose) polymerase-1 deletion and caspase inhibition after stroke. Stroke. (2011) 42:1090–6. 10.1161/STROKEAHA.110.594861 21311064 PMC3066270

[72] Li X Klaus JA Zhang J Xu Z Kibler KK Andrabi SA. Contributions of poly(ADP-ribose) polymerase-1 and−2 to nuclear translocation of apoptosis-inducing factor and injury from focal cerebral ischemia. J Neurochem. (2010) 113:1012–22. 10.1111/j.1471-4159.2010.06667.x 20236222 PMC2860677

[73] Zhang J Li X Kwansa H Kim YT Yi L Hong G. Augmentation of poly(ADP-ribose) polymerase-dependent neuronal cell death by acidosis. J Cereb Blood Flow Metab. (2017) 37:1982–93. 10.1177/0271678X16658491 27381826 PMC5464694

[74] Kofler J Otsuka T Zhang Z Noppens R Grafe MR Koh DW. Differential effect of PARP-2 deletion on brain injury after focal and global cerebral ischemia. J Cereb Blood Flow Metab. (2006) 26:135–41. 10.1038/sj.jcbfm.9600173 15959455

[75] Endres M Scott G Namura S Salzman AL Huang PL Moskowitz MA. Role of peroxynitrite and neuronal nitric oxide synthase in the activation of poly(ADP-ribose) synthetase in a murine model of cerebral ischemia-reperfusion. Neurosci Lett. (1998) 248:41–4. 9665659 10.1016/s0304-3940(98)00224-9

[76] Giovannelli L Cozzi A Guarnieri I Dolara P Moroni F. Comet assay as a novel approach for studying DNA damage in focal cerebral ischemia: differential effects of NMDA receptor antagonists and poly(ADP-ribose) polymerase inhibitors. J Cereb Blood Flow Metab. (2002) 22:697–704. 10.1097/00004647-200206000-00008 12045668

[77] Sairanen T Szepesi R Karjalainen-Lindsberg ML Saksi J Paetau A Lindsberg PJ. Neuronal caspase-3 and PARP-1 correlate differentially with apoptosis and necrosis in ischemic human stroke. Acta Neuropathol. (2009) 118:541–52. 10.1007/s00401-009-0559-3 19529948

[78] Kang HC Lee YI Shin JH Andrabi SA Chi Z Gagne JP. Iduna is a poly(ADP-ribose) (PAR)-dependent E3 ubiquitin ligase that regulates DNA damage. Proc Natl Acad Sci USA. (2011) 108:14103–8. 10.1073/pnas.1108799108 21825151 PMC3161609

[79] Andrabi SA Kang HC Haince JF Lee YI Zhang J Chi Z. Iduna protects the brain from glutamate excitotoxicity and stroke by interfering with poly(ADP-ribose) polymer-induced cell death. Nat Med. (2011) 17:692–9. 10.1038/nm.2387 21602803 PMC3709257

[80] Li X Nemoto M Xu Z Yu SW Shimoji M Andrabi SA. Influence of duration of focal cerebral ischemia and neuronal nitric oxide synthase on translocation of apoptosis-inducing factor to the nucleus. Neuroscience. (2007) 144:56–65. 10.1016/j.neuroscience.2006.08.065 17049179 PMC1876769

[81] Xu Z Zhang J David KK Yang ZJ Li X Dawson TM. Endonuclease G does not play an obligatory role in poly(ADP-ribose) polymerase-dependent cell death after transient focal cerebral ischemia. Am J Physiol Regul Integr Comp Physiol. (2010) 299:R215–21. 10.1152/ajpregu.00747.2009 20427721 PMC2904146

[82] Thorsell AG Ekblad T Karlberg T Low M Pinto AF Tresaugues L. Structural basis for potency and promiscuity in poly(ADP-ribose) polymerase (PARP) and tankyrase inhibitors. J Med Chem. (2017) 60:1262–71. 10.1021/acs.jmedchem.6b00990 28001384 PMC5934274

[83] Takahashi K Greenberg JH. The effect of reperfusion on neuroprotection using an inhibitor of poly(ADP-ribose) polymerase. Neuroreport. (1999) 10:2017–22. 10424667 10.1097/00001756-199907130-00005

[84] Abdelkarim GE Gertz K Harms C Katchanov J Dirnagl U Szabo C. Protective effects of PJ34, a novel, potent inhibitor of poly(ADP-ribose) polymerase (PARP) in *in vitro* and *in vivo* models of stroke. Int J Mol Med. (2001) 7:255–60. 10.3892/ijmm.7.3.255 11179503

[85] Komjati K Mabley JG Virag L Southan GJ Salzman AL Szabo C. Poly(ADP-ribose) polymerase inhibition protect neurons and the white matter and regulates the translocation of apoptosis-inducing factor in stroke. Int J Mol Med. (2004) 13:373–82. 10.3892/ijmm.13.3.373 14767566

[86] Iwashita A Tojo N Matsuura S Yamazaki S Kamijo K Ishida J. A novel and potent poly(ADP-ribose) polymerase-1 inhibitor, FR247304 (5-chloro-2-[3-(4-phenyl-3,6-dihydro-1(2H)-pyridinyl)propyl]-4(3H)-quinazolinone), attenuates neuronal damage in *in vitro* and *in vivo* models of cerebral ischemia. J Pharmacol Exp Ther. (2004) 310:425–36. 10.1124/jpet.104.066944 15075382

[87] Nakajima H Kakui N Ohkuma K Ishikawa M Hasegawa T. A newly synthesized poly(ADP-ribose) polymerase inhibitor, DR2313 [2-methyl-3,5,7,8-tetrahydrothiopyrano[4,3-d]-pyrimidine-4-one]: pharmacological profiles, neuroprotective effects, and therapeutic time window in cerebral ischemia in rats. J Pharmacol Exp Ther. (2005) 312:472–81. 10.1124/jpet.104.075465 15466246

[88] Egi Y Matsuura S Maruyama T Fujio M Yuki S Akira T. Neuroprotective effects of a novel water-soluble poly(ADP-ribose) polymerase-1 inhibitor, MP-124, in *in vitro* and *in vivo* models of cerebral ischemia. Brain Res. (2011) 1389:169–76. 10.1016/j.brainres.2011.03.031 21420942

[89] Moroni F Cozzi A Chiarugi A Formentini L Camaioni E Pellegrini-Giampietro DE. Long-lasting neuroprotection and neurological improvement in stroke models with new, potent and brain permeable inhibitors of poly(ADP-ribose) polymerase. Br J Pharmacol. (2012) 165:1487–500. 10.1111/j.1476-5381.2011.01666.x 21913897 PMC3372732

[90] El Amki M Lerouet D Garraud M Teng F Beray-Berthat V Coqueran B. Improved reperfusion and vasculoprotection by the poly(ADP-Ribose)polymerase inhibitor PJ34 after stroke and thrombolysis in mice. Mol Neurobiol. (2018) 55:9156–68. 10.1007/s12035-018-1063-3 29651748

[91] Teng F Zhu L Su J Zhang X Li N Nie Z. Neuroprotective effects of poly(ADP-ribose)polymerase inhibitor olaparib in transient cerebral ischemia. Neurochem Res. (2016) 41:1516–26. 10.1007/s11064-016-1864-6 26869042

[92] Culmsee C Zhu C Landshamer S Becattini B Wagner E Pellechia M. Apoptosis-inducing factor triggered by poly(ADP-Ribose) polymerase and bid mediates neuronal cell death after oxygen-glucose deprivation and focal cerebral ischemia. J Neurosci. (2005) 25:10262–72. 10.1523/JNEUROSCI.2818-05.2005 16267234 PMC6725791

[93] Berger NA Besson VC Boulares AH Burkle A Chiarugi A Clark RS. Opportunities for the repurposing of PARP inhibitors for the therapy of non-oncological diseases. Br J Pharmacol. (2018) 175:192–222. 10.1111/bph.13748 28213892 PMC5758399

[94] Muscal JA Thompson PA Giranda VL Dayton BD Bauch J Horton T. Plasma and cerebrospinal fluid pharmacokinetics of ABT-888 after oral administration in non-human primates. Cancer Chemother Pharmacol. (2010) 65:419–25. 10.1007/s00280-009-1044-3 19526240 PMC2953793

[95] Donawho CK Luo Y Luo Y Penning TD Bauch JL Bouska JJ. ABT-888, an orally active poly(ADP-ribose) polymerase inhibitor that potentiates DNA-damaging agents in preclinical tumor models. Clin Cancer Res. (2007) 13:2728–37. 10.1158/1078-0432.CCR-06-3039 17473206

[96] Takahashi K Greenberg JH Jackson P Maclin K Zhang J. Neuroprotective effects of inhibiting poly(ADP-ribose) synthetase on focal cerebral ischemia in rats. J Cereb Blood Flow Metab. (1997) 17:1137–42. 9390644 10.1097/00004647-199711000-00001

[97] Takahashi K Pieper AA Croul SE Zhang J Snyder SH Greenberg JH. Post-treatment with an inhibitor of poly(ADP-ribose) polymerase attenuates cerebral damage in focal ischemia. Br Res. (1999) 829:46–54. 10350529 10.1016/s0006-8993(99)01335-9

[98] Yap E Tan WL Ng I Ng YK. Combinatorial-approached neuroprotection using pan-caspase inhibitor and poly (ADP-ribose) polymerase (PARP) inhibitor following experimental stroke in rats; is there additional benefit? Brain Res. (2008) 1195:130–8. 10.1016/j.brainres.2007.12.024 18207135

[99] Haddad M Beray-Berthat V Coqueran B Palmier B Szabo C Plotkine M. Reduction of hemorrhagic transformation by PJ34, a poly(ADP-ribose)polymerase inhibitor, after permanent focal cerebral ischemia in mice. Eur J Pharmacol. (2008) 588:52–7. 10.1016/j.ejphar.2008.04.013 18468597

[100] Teng F Beray-Berthat V Coqueran B Lesbats C Kuntz M Palmier B. Prevention of rt-PA induced blood-brain barrier component degradation by the poly(ADP-ribose)polymerase inhibitor PJ34 after ischemic stroke in mice. Exp Neurol. (2013) 248:416–28. 10.1016/j.expneurol.2013.07.007 23876515

[101] Alkayed NJ Harukuni I Kimes AS London ED Traystman RJ Hurn PD. Gender-linked brain injury in experimental stroke. Stroke. (1998) 29:159–66. 9445346 10.1161/01.str.29.1.159

[102] Alkayed NJ Murphy SJ Traystman RJ Hurn PD Miller VM. Neuroprotective effects of female gonadal steroids in reproductively senescent female rats. Stroke. (2000) 31:161–8. 10.1161/01.str.31.1.161 10625733

[103] Hagberg H Wilson MA Matsushita H Zhu C Lange M Gustavsson M. PARP-1 gene disruption in mice preferentially protects males from perinatal brain injury. J Neurochem. (2004) 90:1068–75. 10.1111/j.1471-4159.2004.02547.x 15312162

[104] Yuan M Siegel C Zeng Z Li J Liu F McCullough LD. Sex differences in the response to activation of the poly (ADP-ribose) polymerase pathway after experimental stroke. Exp Neurol. (2009) 217:210–8. 10.1016/j.expneurol.2009.02.012 19268668 PMC2672307

[105] Liu F Li Z Li J Siegel C Yuan R McCullough LD. Sex differences in caspase activation after stroke. Stroke. (2009) 40:1842–8. 10.1161/STROKEAHA.108.538686 19265047 PMC2674515

[106] Siegel CS McCullough LD. NAD+ and nicotinamide: sex differences in cerebral ischemia. Neuroscience. (2013) 237:223–31. 10.1016/j.neuroscience.2013.01.068 23403179 PMC3609901

[107] Ding Y Zhou Y Lai Q Li J Gordon V Diaz FG. Long-term neuroprotective effect of inhibiting poly(ADP-ribose) polymerase in rats with middle cerebral artery occlusion using a behavioral assessment. Brain Res. (2001) 915:210–7. 10.1016/s0006-8993(01)02852-9 11595210

[108] Sun HS Doucette TA Liu Y Fang Y Teves L Aarts M. Effectiveness of PSD95 inhibitors in permanent and transient focal ischemia in the rat. Stroke. (2008) 39:2544–53. 10.1161/STROKEAHA.107.506048 18617669

[109] Li H Pin S Zeng Z Wang MM Andreasson KA McCullough LD. Sex differences in cell death. Ann Neurol. (2005) 58:317–21. 10.1002/ana.20538 15988750

[110] Takeshima R Kirsch JR Koehler RC Traystman RJ. Tirilizad treatment does not decrease early brain injury after transient focal ischemia in cats. Stroke. (1994) 25:670–6. 8128524 10.1161/01.str.25.3.670

[111] Takeshima R Kirsch JR Koehler RC Gomoll AW Traystman RJ. Monoclonal leukocyte antibody (MoAb 60.3) does not decrease injury following transient focal cerebral ischemia in cats. Stroke. (1992) 23:247–52. 1561656 10.1161/01.str.23.2.247

[112] Rebel A Ulatowski JA Joung K Bucci E Traystman RJ Koehler RC. Regional cerebral blood flow in cats with cross-linked hemoglobin transfusion during focal cerebral ischemia. Am J Physiol Heart Circ Physiol. (2002) 282:H832–41. 10.1152/ajpheart.00880.2001 11834476

[113] Maier C Scheuerle A Hauser B Schelzig H Szabo C Radermacher P. The selective poly(ADP)ribose-polymerase 1 inhibitor INO1001 reduces spinal cord injury during porcine aortic cross-clamping-induced ischemia/reperfusion injury. Intensive Care Med. (2007) 33:845–50. 10.1007/s00134-007-0585-3 17361386

